# Caesarean Section vs. Normal Vaginal Delivery: A Game Theory Discussion in Reimbursement Interventions

**Published:** 2018-11

**Authors:** Marita MOHAMMADSHAHI, Hasan HEMATYAR, Masoumeh NAJAFI, Minoo ALIPOURI SAKHA, Abolghasem POURREZA

**Affiliations:** Dept. of Health Management and Economics, School of Public Health, Tehran University of Medical Sciences, Tehran, Iran

**Keywords:** Caesarian section, Normal vaginal delivery, Game theory, Insurance

## Abstract

**Background:**

The rate of caesarean section (C-section) in Iran is too high, so having a plan to control it is crucial. Since one of the most important reasons for inclination of providers to do C-section is financial issues, the purpose of this study was offering financial solutions for increasing normal vaginal delivery (NVD) and decreasing non-indicated C-section.

**Methods:**

This analytical-descriptive research, used game theory for offering financial mechanisms. The game was a dynamic one in which the backward induction was used to obtain a Nash equilibrium. Financial structure and the mean number of NVD and C-section in a certain period of time in comparison with standards were as the main influential factors on financial dimensions and were included in the model.

**Results:**

The effect of financial structure was shown through a specified insurance for childbirth, existence of a monitoring department and tariffs.

**Conclusion:**

The main solution for controlling C-section in designed game was taxes and fines for physician or hospital in non- indicated cases and giving reward otherwise.

## Introduction

Over the past two decades, C-section rate in developed and developing countries has been increasing. The main reason is unnecessary and elective C-section by patients and their physicians (1, 2) which lead to problems for mother, her infant; and economic burden and incremental costs for households and society (3–5). The mean cost of services in each C-section was significantly more than NVD [6]. In fact cost of C-section, time duration of hospitalization, recovery, and return to work is two times more than NVD (7). There is no justification to have deviation from 15% revalence for C-section [8, 9]. However, this procedure has been done more than standard in many countries like the United State, Norway, Scotland and Sweden (2, 10, 11).

In Iran, the rate of C-section is between 26%–66.5% and in private sectors it can be about 87% (8, 12, 13). According to the report of Ministry of Health in 2011, 1.3 million childbirth were registered that about 53% were done in C-section. WHO report in 2010 showed that Iran with 41.6% C-section had the second rank in the world (14).

Due to the uncontrolled increase in the rate of C-section in Iran, having a plan for managing this procedure is too important. One of the solutions to create tendency in providers to promotion NVD is adjusting tariffs and payment system because financial issues are one of the reasons for doing C-section (15, 16). Therefore, the scope of this study was on the financial dimension of childbirth and we tried to offer a financial mechanism for decreasing C-section.

Undoubtedly, income is an important source of motivation in the behavior of providers. We emphasized on supply side or behavior of providers and their tangible motivation (income) have been investigated by designing a model in game theory context to access to WHO standard.

## Methods

The present study employed an analytical-descriptive research method. We used game theory to render solutions for financial reforms. Game theory assumes the human social behaviors and actions are calculated and it considers the human being as a rational (cost-benefit calculation) creature. This theory was first introduced in 1921, and afterward, other theorists began to develop the idea (17).

Each of the games has rules and structures according to which the players reach their goals. The rules and structures define the reasons and actions of each player. In the real world, people's decision making is accompanied by others' reactions. The payoffs for each situation depend upon his own decisions as well as the decisions of others (18).

The games can be classified in a number of ways such as static and dynamic, based on the number of players, the number of game strategies (finite and infinite), the nature of the payment function (zero- or non-zero-sum games) and based on the characteristics of the negotiations before the game (aligned or non-aligned.) The static games are solved using dominant strategies and weakly dominated strategies, mixed strategy equilibrium, minimax method and Nash equilibrium.

In dynamic games discussed in this article, players make sequential decisions, know the game's history and are also aware of the payoffs to every strategy. These games are usually represented by extensive form for analysis and description and game trees. The payoff to every player is indicated in the last branch of the game tree associated with every strategy. Rationality is ruling the game and the players have complete knowledge and awareness. In dynamic games, since the player may choose from many decision nodes, a pure strategy in this game helps the player decide what action to take at any information sets. Dynamic games can be solved in various ways. This paper employs the backward induction method to obtain the Nash equilibrium in the game. A Nash equilibrium is a set of strategies, one for each player, such that given the strategies being played by others, no player can improve on his payoff by adopting an alternative strategy (19). To find a solution using the Nash equilibrium, first it must be written in the strategic form and then the Nash equilibrium should be found as in static games. Equilibrium in dynamic games of complete and perfect information is known as backward induction Nash equilibrium. In such equilibrium, the player begins at the terminal nodes associated with a single information set. For the decision node, the decision-maker chooses a branch to take which has the highest payoff among the branches emerging from that node and ending in the terminal node, every branch indicating a player's action. The taken branch can be part of the whole game equilibrium if it is part of the continuous path connected to the first node.

## Results

In discussing financial solutions to reduce C-section rate and to increase NVD rate, factors contributing to the financial aspect used in the model are as follows:

a) Financial structures: several financial factors have contributed to the increasing willingness of providers to do C-section. These are as follows:
  1) Independent and unified insurance to pay for childbirth: Reforming the insurance structures in Iran will be definitely very helpful. The formation of independent and unified insurance system covering the childbirth costs has many benefits. Here, we briefly refer to some of the benefits:
    ➢ A single buyer raises the power of bargaining with providers, therefore, improves the quality of the providers' performance.
    ➢ A single dominant service buyer has the power to compel the providers to reduce rates of unnecessary C-section, and fine them if the providers do not meet the standards.
    ➢ The existence of an independent service buyer increases the power of winning the proceedings which lead to the increased rates of NVD.
    ➢Insurance costs, particularly costs associated with court proceedings, will be greatly reduced because of aggregation childbirth insurance.
  2) A monitoring unit: the presence of a supervisor to monitor the performance of gynecologists can avoid unnecessary C-sections. The most appropriate supervisor is the insurance fund that, being a beneficiary, is able to reduce unnecessary C-sections by attending the childbirth procedures.
  3) Tariffs of C-section and normal delivery: In Health Reform Plan in Iran, surgery k coefficient was calculated 40k for caesarean and 50k for normal delivery; however, physicians have no tendency to perform natural birth yet. The reasons for the absence of tendency include time-consuming nature of childbirth and the risks of NVD are relatively higher than C-sections and we should consider opportunity-cost of physicians.

If P_1_ was the price received for NVD and n_1_ was the mean NVD rate of a period, taking into account that the expert only performs NVD, minimum value of p_1_ × n_1_ must be equal to p_2_ × n_2_, in which p_2_ is the price received for C-section by the expert and n_2_ is the average number of C-sections in a period. When an expert only performs C-sections, p_1_ ≥ n_1_× n_2_× p_2_ in which p_1_> p_2_ since n_2_> n_1_ and this means an increased price for NVD. Any change in the price that does not fulfill the above-mentioned condition is a fiscal policy doomed to failure. Failure to achieve the above equation is the real cause of physicians' tendency to practice C-sections despite the changes in surgery coefficient k.

**Fig. 1:**
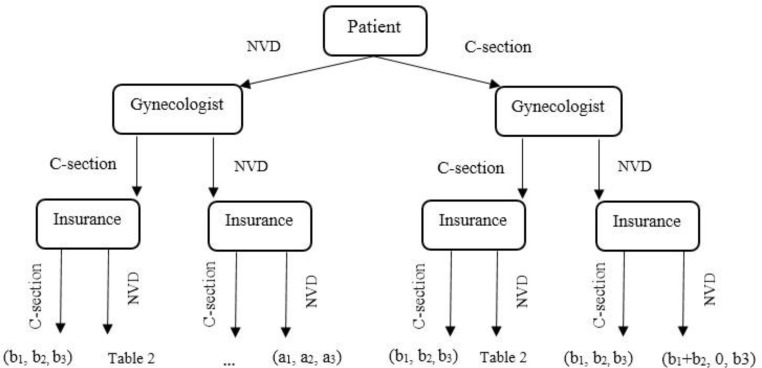
Childbirth procedure and its payoffs

The process of making the financial payoffs of childbirth procedure is based on expert opinion, and data gathered through questionnaire. Therefore, we have developed the following diagram to show the financial payoffs of childbirth procedure:

In the childbirth procedure, there are many decision-makers and beneficiaries consisting of the patient, physician and third-party (as the control). In Fig. 1, the childbirth procedure and its payoffs are shown. In this diagram a_1_ is client’s payment for NVD, b_1_ is payment for C-section, a_2_ is insurance rate for NVD, b_2_ is insurance rate for C-section, a_3_ is physician earnings for NVD and b_3_ is physician earnings for C-section. We treated the game as a dynamic game of complete information in this modeling. We investigate all the issues from the viewpoints of player one (physician) and also player two (insurance agent).

**Fig. 2:**
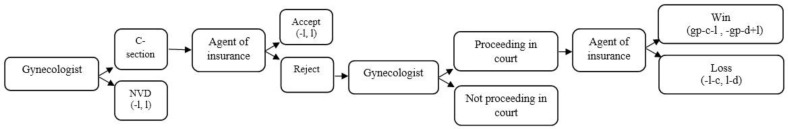
The analysis framework of the game

General premises and game expansion: the analysis framework of the game is included in Fig. 2. The first move in this game is done by the gynaecologist. Suppose the physician earns a dollars for a NVD and b dollars for a C-section operation a<b, and b−a=1, then 1 is the interest of C-section compared with the NVD. If the physician recommends NVD to someone and do it, the insurance gains 1 dollar, therefore, the payoffs of the physician and insurance will be [−1, 1] respectively.

Where (c) is the opportunity cost of the physician in court proceedings, (d) is the opportunity cost of the insurance in court proceedings, (g) is suggested index while negotiating, l is C-section and normal vaginal delivery difference rate, (p) is the price of physician’s proceedings and (q) is the possibility of the physician winning the proceedings in court.

Suppose that the physician recommends C-section. In that case, this is the agent’s turn. The insurance agent can endorse caesarean or reject it if regards it as unnecessary. In case of endorsement of the physician’s opinion of C-section by the insurance agent, the payoffs of the physician and insurance will be [−1, 1], respectively. However, if the agent rejects the C-section necessity, the next move is done by the physician.

The physician can proceed against the insurance or decline the proceedings. If the gynaecologist declines the proceedings, he will receive the payment for a NVD, hence the payoffs will be as [−1,1]. However, if he proceeds, the third physician checks the proceedings (Fig. 2).

If the third physician confirms the C-section (he will win the court while the probability of failure is (q), he will receive p dollars from the insurance. The physician’s payoff will be p-c-l in case of winning the court where c represents the physician’s opportunity cost during the legal procedure. The significant point here is that the physician should always notice the payoff of the proceedings which disjoints the relationship between him and the insurance and downgrades the physician’s professional position, in addition to the attorney, legal or other fees. Therefore, as we calculate the opportunity cost (c), the indirect costs consisting of detrimental effects on the physician’s future reputation should be taken into account along with the consideration of direct costs.

The physician gathers his documents and intends to proceed against the insurance for p Rials. In this case, the insurance has two options. It can bargain with the physician and proposes a percentage of contracts, for instance gp Rials in which g is between 0 and 1. On the other hand, the insurance payoff in case of the physician’s win will be p−d+l in which d includes the opportunity cost of insurance attending the court. In order to calculate d similar to that of c, the indirect costs are considered.

### Investigating the equilibrium in the game:

To solve the game, first, we solve the diagram in Fig. 2 from the end to the start step by step while considering parties’ payoffs: The expected payoff of the proceedings is calculated by the following equation 1 (with the risk of neutrality of players) (Fig. 2):

eq.1 (q(p−c−1)+(1−q)(−c−1),q(−p−d+d)(1−q)(−d+1))=(qp−c−1,−qp−d+1)

Where the expected insurance payoff is −qp-d+1 and the expected physician payoff is qp-c-1. The physician will file a complaint when its payoff is higher than or equal to the absence of it. Therefore, the condition for a complaint done by the physician is as following equation 2:

eq.2 qp−c−1≥−1,  qp≥c

**Fig. 3:**
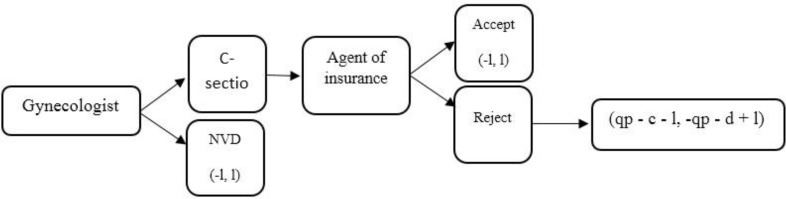
Condition for a complaint done by the physician

This is shown in Fig. 3.

The equilibrium path, here, means a path in which the game tree is traced at the equilibrium points, and the path in each sub-game is unique.

b) Considering the mean NVD and C-section rate in a certain time and comparing them with the standards: to prevent unnecessary C-sections, we can use several solutions in the game:

1) Taxing or fining physicians who performing unnecessary C-sections: each physician should pay tax on a stepped-up basis. Table 1 is the stepped-up basis table for taxing the physicians which leads to a decreased C-section by fining the physician. The gained taxes could be used as a source of financing for NVD development.
2) Fining the hospital or medical units that perform unnecessary C-section. This fine can be executed financially similar to the above table for hospitals or executed by downgrading the accreditation of the hospital. These levers of control cause higher supervision of the hospital on the maternity ward.

## Discussion

The present study aimed to propose financial patterns and corrective strategies to promote NVD and reduce unnecessary C-sections in Iran by using the game theory method.

**Table 1:** Stepped-up taxing or fining the physicians who performing unnecessary C-sections

***Normal vaginal delivery per 100 childbirth (m)***	***Caesarean section per 100 childbirth (n)***	***Income of gynecologist***
100 - 40	0 - 60	nb_3_ + ma_3_
40 - 30	60 - 70	%80nb_3_ + ma_3_
30 - 20	70 - 80	%60nb_3_ + ma_3_
20 - 10	80 - 90	%40nb_3_ + ma_3_
10 - 0	90 - 100	%20nb_3_ + ma_3_

Note: n: Caesarean section per 100 childbirth, m: Normal vaginal delivery per 100 childbirth

a3: physician earnings for NVD, b3: physician earnings for C-section

The increasing trend of cesarean in the world and especially in Iran has imposed huge costs on health systems. Various methods have been implemented in different countries to control and reduce the rate of C-sections. Some of these methods such as preparation of clinical guidelines, obligation of getting advice from another expert, monitoring and scrutiny before any potential cesarean, counseling before a C-section, and holding antenatal preparation classes in low-risk pregnancies have almost been successful in reduction of cesarean rate (20).

In hospitals that compete with each other at a medium level, the cost of NVD was very lower, while in hospitals that wages and case mix index (DRGs) were higher, the payment was much higher. In hospitals which were operating in a competitive market, the payment was much lower for people who were uninsured while in for-profit hospitals and where higher wages are paid, the payment was much higher (21).

The costs of C-section and NVD were studied and its major factors in the health system of a series of countries with upper-middle income. The average direct cost per case during hospitalization for C-section group (about $96,992) was significantly more than those with NVD (about $96,992) (22).

Physician decisions could be affected by both monetary and non-monetary incentives and the perception of the patient’s medical information and preferences. Non-clinically appropriate C-section occurred at a moderate but growing rate of roughly 3.2% in New Jersey over the period 1999–2002, in which explained mainly by non-clinical factors (23).

Revision of childbirth tariffs and payment system seems to be one of the ways to create a desire for providers to promote NVD, because monetary issues are one of the main reasons for willingness of providers to perform cesarean. Currently, in Iran, 86% of deliveries are conducted in public medical-training hospitals where the modern hospital system is running. More earnings will be achieved by performing more procedures. This has increased the willingness of most gynecologists to perform cesarean (14). The effect of system of payment was studied in public and private hospitals on the rate of C-section. There was a fixed system of payment in public hospitals, while in private hospitals fee for service is regular. There was no difference in the income of physicians in public hospitals if he/she performed a cesarean, while it had a positive impact on physicians’ income in private hospitals. The likelihood of C-section in the private sector is almost 2 times more than the public sector. Cesarean frequency in women without medical indication is 11% in public hospitals and 24% in private hospitals. The system of payment to physicians is one of the main causes of these differences (24).

Regarding the tariff change, the therapists and insurance organizations are the major actors in childbirth. Hence, these two groups have been emphasized to adjust the financial model in this study. The adopted financial policies based on increasing *K* coefficient of NVD and fee-for-service methods for both NVD and C-section not only have created enough motivation for providers and insurance agencies for controlling C-section but also have not established a reasonable and desirable balance between them. The reason for lack of welcome of these financial policies by the major actors has been basically related to ignoring the opportunity costs of service providers to perform C-section or NVD. However, this is due to several reasons, the most important are organization of programs and decisions and structural failures of the health system in decision-making. The scientific bases of this policy that increasing *K* coefficient alone can control cesarean rate was not clear to us. Even if this policy is evidence-based, it is a one-dimensional and incomplete policy which fails to take into account C-section or NVD as a whole phenomenon with all its dimensions and active actors. Since financial dimension is only one of multiple dimensions affecting the demand and supply for childbirth, financial reforms alone cannot be considered the best way of reducing the rate of unnecessary C-sections. For making reforms in any field, all factors including structural, legal, sociocultural, and financial factors should be simultaneously taken into account. In China, part of the high rate of C-section was related to social and cultural factors. China’s medical system is highly dominated by the preferences of service users. The current payment system and bonus collection system for physicians are other factors affecting the increased rate of C-section. In order to control labor costs, China adopted the policy of price transparency to oblige hospitals to declare the average amount of money received from patients. However, the policy failed (25).

Referral of all deliveries to Gynecologists regardless of the performance of midwives naturally increases the demand and raises labor costs. With regard to the time limits and increased volume of demand and also the fact that pursuit of interests is part of human nature, therapists follow alternative ways to make greater use of their time and skills. In this regard, the opportunity cost of labor help to increase the cesarean rate. This means that, even without indication, cesarean is not accessible with making a change to payment methods, although it may temporarily affect each of these two types of delivery.

## Conclusion

The mechanisms used for controlling C-sections, especially without indication include integration of maternity insurance. This means that only one insurance institution to be the buyer and payer of services. If the structure of such insurance is scientifically developed, considering the high power of bargaining and price control, it could be efficient. However, some mechanisms can be designed within this insurance to maximize the income of physicians who comply with the norms of the health system and apply the standards introduced by the Ministry of Health or fine those who violate these rules. This can be a source of funding for NVD costs. In addition, the hospitals that comply with these norms can be subject to such rules in the financial and accreditation fields. Another mechanism applied by insurance companies is the certificate of need for C-section prior to performing it. This mechanism can also be helpful in the control of costs. In fact, development of a scientific and firm structure on the one hand and monitoring of administrative processes, on the other hand, can be helpful in achieving the goals in relation to increasing the frequency of NVD.

## Ethical considerations

Ethical issues (Including plagiarism, informed consent, misconduct, data fabrication and/or falsification, double publication and/or submission, redundancy, etc.) have been completely observed by the authors.
